# Novel heterozygous mutations of SLC12A3 gene in a Chinese pedigree with Gitelman syndrome: A care-compliant case report

**DOI:** 10.1097/MD.0000000000034967

**Published:** 2023-09-01

**Authors:** Ye Bi, Ming-Yang Kuang, Ming-Long Li

**Affiliations:** a Department of Geriatric Endocrinology, Shandong Provincial Hospital Affiliated to Shandong University, Jinan, China.

**Keywords:** c.2089_2095del (exon17), c.857 A >C (exon7), Gitelman syndrome, hypokalemia, hypomagnesaemia, SLC12A3 gene

## Abstract

**Rationale:**

The diagnosis of Gentleman syndrome (GS) is usually delayed because the clinical symptoms are easily mistaken.

**Patient concerns:**

A 19-year-old male patient was referred to endocrinology due to intermittent twitch of extremities for approximately 7 years.

**Diagnoses:**

The diagnosis of GS was made based on the laboratory and gene detection results. We identified 2 new variants in the SLC12A3 gene [c.857 A > C (exon7) and c.2089_2095del (exon17)] in his Asian family.

**Interventions:**

The patient received the treatment of potassium chloride sustained release tablets, potassium magnesium aspartate and spironolactone. After given potassium supplement through enema, his serum potassium level was corrected to normal.

**Outcomes:**

The electrolyte imbalance including hypokalemia and hypomagnesemia were improved with a remission of the clinical manifestations. But the patient’s condition still could not remain stable for his irregular oral potassium supplementation during the follow-up of nearly 3 months.

**Lessons:**

Our finding broadens the variant spectrum of SLC12A3 and contributes to a more quickly genetic counseling. As a result, when a patient presents with persistent, unspecified, and inadequately treated hypokalemia, tests for GS should indeed be considered. For suspected cases of GS, genetic testing should always be considered in the diagnosis.

## 1. Introduction

Gitelman syndrome (GS) is a tubulopathy characterized by hypokalemic metabolic alkalosis in combination with significant hypomagnesemia and hypocalciuria, which is an autosomal recessive disease primarily caused by mutations in SLC12A3 gene.^[1]^ GS is one of the most common causes of inherited hypokalemia and is a highly heterogeneous disease.

## 2. Case report

Mutations in the SLC12A3 gene have been reported to cause GS. Here, we investigated the mutation of SLC12A3 gene in a Chinese pedigree with GS and analyzed the clinical manifestations. This study were reviewed and approved by the ethics committee of Shandong Provincial Hospital. Written informed consent was obtained from the individual(s).

The proband, a 19-year-old male patient was hospitalized because of intermittent twitch of extremities for approximately 7 years. At the age of 12, the patient suddenly suffered from expiratory dyspnea and tetany after coma treated with Qing-Kai-Ling injection. He once visited the local hospital and found decreased plasma potassium. Since that episode, he presented recurrent twitch over all extremities, often induced by diarrhea or emotional factors. Further diagnosis was not made and he felt better after treatment with “potassium chloride”, but experienced recurrence and aggravation of these symptoms. However, his serum potassium level decreases gradually, usually to 2.6 to 2.7 mmol/L, was difficult to correct to a normal range. Two weeks prior, decreased muscle strength in extremities accompanied by palpitation and dyspnea recurred after sweating and the patient was unable to stand and walk, consequently he visited the local hospital. He was diagnosed as “hypokalemia” and the lowest potassium level in blood test was 2.4 mmol/L. His symptoms were improved after receiving oral potassium but potassium level was only raised to 2.52 mmol/L.

During examination, the patient was conscious. Physical examination on admission showed the temperature of 36.0°C, heart rate of 90 beats per minute, respiratory rate of 18 breaths per minute, and blood pressure of 110/67 mm Hg. He weighed 81 kg with a height of 178 cm, and his body mass index was 25.56 kg/m^2^. No abnormalities were identified in muscle strength and muscle tone and the physical examination of the lung, cardiac, and abdomen. Laboratory findings of patients suggested severe hypokalemia, hypomagnesemia, hypocalciuria (urine calcium/creatinine is 0.04), metabolic alkalosis, and secondary hyperaldosteronism (Table 1). The electrocardiogram showed tachycardia with ST-T changes. The family history indicates that the patient’s parents are not consanguineous. His twin brother had more slight symptoms of occasional weakness than him with mild hypokalemia and hypocalciuria and mild hypomagnesemia. All other pedigree members did not have clinical symptoms related to GS.

**Table 1:** Biochemical characteristics of the proband.

Examination item	Test value	Reference value
Serum biochemicals		
Potassium (mmol/L)	2.4	3.5–5.5
Sodium (mmol/L)	141.1	137–147
Chloride (mmol/L)	96.9	99–110
Calcium (mmol/L)	2.44	2.2–2.7
Phosphate (mmol/L)	1.08	1.29–2.26
Magnesium (mmol/L)	0.69	0.75–1.02
Creatine (μmol/L)	60.26	40–135
BUN (mmol/L)	6.1	2.8–7.14
CO2CP (mmol/L)	35.9	22–29
Urine test		
Urine specific gravity	1.022	1.003–1.030
PH	7.5	5–7
24 h Urine tests		
Potassium (mmol/24 h)	69.7	51–100
Calcium (mmol/24 h)	0.71	2.5–7.5
Magnesium (mmol/24 h)	5.02	3–5
Chloride (mmol/24 h)	433.5	280–420
Sodium (mmol/24 h)	450.5	130–240
Renin-angiotensin–aldosterone system		
Renin (pg/mL)	208.53	4–24
Angiotensin II (pg/L)	111.51	25–129
Aldosterone (pg/dL)	169.26	16–160

**Figure 1.**
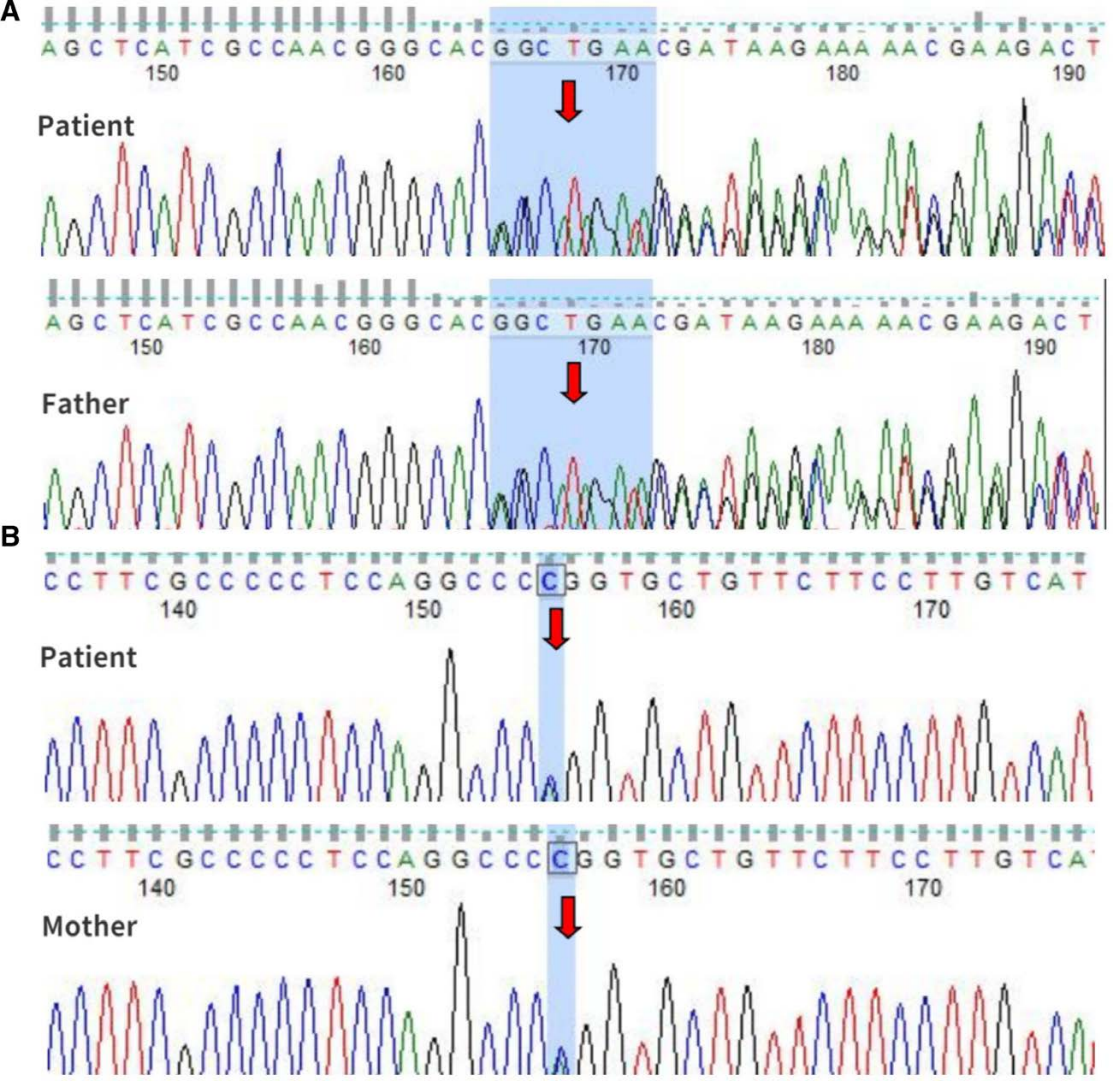
Sequencing peaks for SLC12A3 gene. (A) Gene location (exon number Exon 17, cDNA level 2089_2095del, protein level Thr697fs) is a suspicious disease-causing gene derived from the father. (B) Gene location (exon number exon 7, cDNA level 857 A > C, protein level Gln286Pro), considered to be the disease-causing gene from the mother.

**Figure 2.**
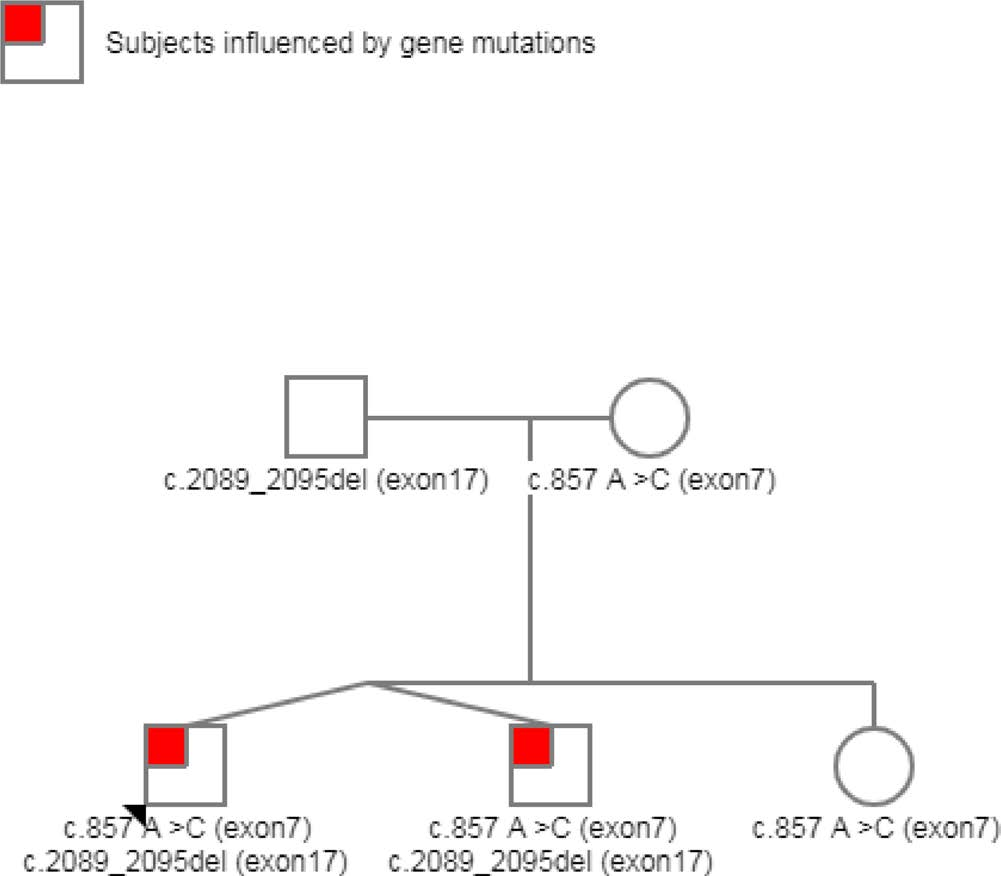
Pedigree of the GS family. Males and females are indicated by squares and circles, respectively. The proband is indicated by an arrow. The patients are represented by a red symbol. GS = Gitelman syndrome.

To confirm the diagnosis of the proband and to screen for the SLC12A3 gene mutation in this pedigree, we performed SLC12A3 gene mutation tests in the 5 family members. As shown in Figure 1 and Figure 2, 2 new heterozygous mutations in the SLC12A3 gene were confirmed: the classic splice-site mutation of c.857 A > C (exon7) which causes amino acid p.Gln286Pro (the patient and his mother and elder sister, twin brother) the frame shift mutation of c.2089_2095del (exon17) which causes amino acid p.Thr697fs (the patient and his father, twin brother). Based on the laboratory and gene detection results, the diagnosis of GS was made.

## 3. Treatment

After the clinical diagnosis was confirmed, the patient received the following treatment: potassium chloride sustained release tablets (6 g/day), potassium magnesium aspartate (6 tablets/day) taken orally 3 times daily and spironolactone (40 mg/day). After the treatment, his twin brother’s serum potassium level increased but remained at the lower limit of normal range and symptoms improved. But unfortunately, the patient did not pay attention to the treatment. He took the medicines irregularly. Two months after discharge, he went to another hospital for twitch of extremities after diarrhea. He was given potassium supplement through enema and then his serum potassium level was corrected to normal. But after that, his condition still could not remain stable for his irregular oral potassium supplementation during the follow-up of nearly 3 months.

## 4. Discussion

Hypokalemia is the most common electrolyte abnormality observed in clinical practice and is found in approximately 20% of hospital inpatients. Severe or prolonged potassium depletion can result in glucose intolerance and serious cardiac, renal, and neurologic dysfunction, including death. Hypokalemia can be effectively corrected if detected in an incipient stage. However, patients with unexplained hypokalemia are a considerable challenge for diagnosis.

GS is inherited tubulopathies with challenges in its precise diagnosis since both pathologies have overlapping clinical symptoms. Regarding GS, it used to be considered as a mild disease, but patients frequently report extreme fatigue, showing an impaired quality of life. This case helps to highlight the fact that early genetic testing is useful to help secure an accurate diagnosis in a timely fashion, as the condition can be difficult to diagnose clinically. This then allows further optimization of treatment, screening of family members and gives the patient a greater understanding of their condition. Our patient remained symptomatic despite optimal potassium and magnesium replacement, and the introduction of potassium supplement through enema did confer some clinical benefit.

GS is an autosomal recessive hereditary disorder characterized by hypokalemic metabolic alkalosis, hypomagnesemia, and hypocalciuria,^[2]^ due to inactivating mutations in the solute carrier family 12, member 3 (SLC12A3) gene that encodes the thiazide-sensitive Na-Cl cotransporter.^[3]^ GS was previously considered as a rare disease, with a reported incidence of approximately 1/50,000. More than 600 different mutations in SLC12A3, including nonsense, splice-site, and missense mutations, have been linked to GS.^[4]^ Mutations in the SLC12A3 gene cause structural changes and/or dysfunction of the renal thiazide-sensitive sodium–chloride cotransporter (NCCT), which leads to disturbance of tubular reabsorption of sodium and chloride ions. Compound heterozygous mutation is more common than homozygous mutation, and no hot spot mutation has been identified yet. Majority of patients have 2 different mutations in both alleles and this site showed the significant diagnostic value of GS in a suspected population. According to domestic reports, T60M, D486N, R913Q and R928C are the most common amino acid mutations in the Chinese population.^[5]^ Clinical symptoms of GS are wide-ranging, from asymptomatic to mild symptoms of fatigue, nocturia, muscle weakness, muscle cramps, and severe symptoms, such as tetany, paralysis, rhabdomyolysis, or lethal arrhythmia. Although GS is described as an asymptomatic or benign disorder, it may develop life-threatening complications, such as ventricular arrhythmia, in some cases.^[6]^ Early detection with suitable treatment may prevent potentially dangerous complications. GS patients might have no symptoms or complaint about decreased muscle strength. Patients with GS are managed with liberal salt intake, potassium, and magnesium supplementation. In case of persistent hypokalemia, potassium-sparing diuretics, renin-angiotensin system blockers, or NSAIDs may be useful. In a study carried out in 147 carriers in the Amish population,^[7]^ lower blood potassium values were associated with heterozygous carriers. It is important to note that all the current treatments are supportive, and currently there is still debate on different topics. There is no consensus on the treatment of practically asymptomatic patients with GS^[8]^ or the definition of the threshold that symptomatic patients with salt-wasting tubulopathy must reach to ensure a good prognosis.^[9]^ Furthermore, the degree of hypokalemia does not totally explain the severity of fatigue in GS patients and polyuria is usually absent or mild.^[10]^

This study described a new compound heterozygous mutation. At present, it is still difficult to determine the correlation between the phenotype and genotype of GS. In general, the symptoms are more severe in homozygous than heterozygous mutations, and worse in male patients compared with female patients.^[11]^

Therefore, the diagnosis of GS should combine the clinical manifestations and laboratory examination with gene analysis, and we recommend all suspected patients undergo gene diagnosis. Nonetheless, the lack of hotspot mutations and the high cost of gene analysis have brought great challenges to the widespread application of this technique. It is crucial for GS patients to be diagnosed early and treated properly. Although we found that the 2 mutations were automatically predicted to be disease-causing mutations, the underlying pathophysiologic mechanisms remains unclear. It is important to find the mechanism by which the compound heterozygous mutations of SLC12A3 gene interact to cause disease. A deeper understanding of the mechanisms involved in the variability and complexity of GS is critical for a better treatment. In addition, hypomagnesemia and hypocalciuria were classical features of patients with GS. Many clinical reports have demonstrated that the function-loss mutations in the SLC12A3 gene, mainly including Thr60Met, Asp486Asn, Gly741Arg, Leu859Pro, Arg861Cys, Arg913Gln, Arg928Cys, and Cys994Tyr, play the pathogenic effects in GS.^[12]^ Our findings demonstrated that administration of using enema in GS patients may reduce the incidence of hypokalemia. The mainstay of treatment remains a lifelong high potassium, magnesium and sodium replacement. However, persistent hypokalemia was observed during outpatient follow-up. Though intermittent intravenous administration can achieve symptom relief, the effect is often short lived and this approach is usually limited by patient acceptability and the burdens of achieving intravenous access. Requirements for potassium and magnesium treatment and replacement are variable, and dosages must be individually tailored and can be very high and still not fully normalize serum biochemistry. This tablet burden can be burdensome for patients.

Unfortunately, our study has several limitations. First of all, the patient took medicines irregularly and long-term follow-up is really needed. Moreover, the treatment data of enema potassium supplement was not detailed. Besides, for not have clinical symptoms related to GS, some persons in this family were not performed SLC12A3 gene mutation tests.

## 5. Conclusion

Here, we report a case of new heterogeneous mutations of SLC12A3 in an Asian pedigree and analyzed the clinical manifestations, laboratory results and genetic features. Our findings strongly suggested that the 2 novel mutations in the SLC12A3 gene are the causative agents of GS, which may provide further insights into the function of this gene and help clinician understand this disorder. Genotype and phenotype vary significantly among GS patients. Further larger population scale studies as well as molecular studies are required to further elucidate the complexity of GS, and to find a better treatment regimen. GS requires lifelong treatment, and regular follow-up is important to prevent advanced-stage chronic kidney disease. The early diagnosis and treatment of this condition can help prevent potentially life-threatening conditions.

## Author contributions

**Conceptualization:** Ye Bi, Ming-Yang Kuang, Ming-Long Li.

**Data curation:** Ye Bi, Ming-Yang Kuang.

**Funding acquisition:** Ming-Long Li.

**Formal analysis:** Ye Bi.

**Investigation:** Ye Bi.

**Methodology:** Ye Bi, Ming-Yang Kuang.

**Supervision:** Ye Bi, Ming-Yang Kuang.

**Writing – original draft:** Ye Bi.

**Writing – review & editing:** Ye Bi, Ming-Yang Kuang, Ming-Long Li.
